# High Cortico-Trabecular Transitional Zone Porosity and Reduced Trabecular Density in Men and Women with Stress Fractures

**DOI:** 10.3390/jcm10051123

**Published:** 2021-03-08

**Authors:** Afrodite Zendeli, Minh Bui, Lukas Fischer, Ali Ghasem-Zadeh, Wolfgang Schima, Ego Seeman

**Affiliations:** 1 Medical Department of Internal Medicine, Rheumatology and Endocrinology at Krankenhaus Herz Jesu, 1030 Vienna, Austria; 2 Centre for Epidemiology and Biostatistics, Melbourne School of Population and Global Health, University of Melbourne, Melbourne 3000, Australia; mbui@unimelb.edu.au; 3 Computational Imaging Research Lab, Department of Biomedical Imaging and Image-Guided Therapy, Medical University of Vienna, 1090 Vienna, Austria; lukas.fischer@scch.at; 4 Software Competence Center Hagenberg GmbH (SCCH), 4232 Hagenberg, Austria; 5 Departments Medicine and Endocrinology, Austin Health, University of Melbourne, Melbourne 3084, Australia; alig@unimelb.edu.au (A.G.-Z.); egos@unimelb.edu.au (E.S.); 6 Department of Diagnostic and Interventional Radiology–Barmherzige Schwestern Krankenhaus, Goettlicher Heiland Krankenhaus und Sankt Josef Krankenhaus, 1060 Vienna, Austria; wolfgang.schima@khgh.at

**Keywords:** bone microstructural deterioration, cortical porosity, high resolution peripheral quantitative computed tomography, stress fracture

## Abstract

To determine whether stress fractures are associated with bone microstructural deterioration we quantified distal radial and the unfractured distal tibia using high resolution peripheral quantitative computed tomography in 26 cases with lower limb stress fractures (15 males, 11 females; mean age 37.1 ± 3.1 years) and 62 age-matched healthy controls (24 males, 38 females; mean age 35.0 ± 1.6 years). Relative to controls, in men, at the distal radius, cases had smaller cortical cross sectional area (CSA) (*p* = 0.012), higher porosity of the outer transitional zone (OTZ) (*p* = 0.006), inner transitional zone (ITZ) (*p* = 0.043) and the compact-appearing cortex (CC) (*p* = 0.023) while trabecular vBMD was lower (*p* = 0.002). At the distal tibia, cases also had a smaller cortical CSA (*p* = 0.008). Cortical porosity was not higher, but trabecular vBMD was lower (*p* = 0.001). Relative to controls, in women, cases had higher distal radial porosity of the OTZ (*p* = 0.028), ITZ (*p* = 0.030) not CC (*p* = 0.054). Trabecular vBMD was lower (*p* = 0.041). Distal tibial porosity was higher in the OTZ (*p* = 0.035), ITZ (*p* = 0.009), not CC. Stress fractures are associated with compromised cortical and trabecular microstructure.

## 1. Introduction

Stress fractures are commonly the result of repetitive loading and are seen in athletes, military recruits and professional dancers, but stress fractures also occur in recreational athletic individuals as vigorous exercise increases the risk for injuries [1,2,3,4,5]. These fractures exist across a spectrum from low-grade stress reactions, bone marrow edema to complete fractures visible radiologically [6,7,8]. Despite there being little or no deficit in areal bone mineral density (aBMD) reported in most studies, there is evidence suggesting that abnormalities in bone microarchitecture are associated with these fractures and so may contribute to bone fragility [6,9,10,11,12,13,14,15,16,17].

As stress fractures are commonly reported in young adults abnormalities in bone morphology associated with these fractures are likely to be due, in part, to abnormalities in the growth and development of peak bone macro- and microstructure, not necessarily bone loss. In the diaphysis, a region composed virtually exclusively of cortical bone, growth in size and mass occurs by periosteal apposition with concurrent endocortical resorptive modeling producing a radial ‘modeling drift’ so the cortex becomes thinner relative to its increasing total cross-sectional area (CSA) [18,19].

By contrast, the metaphyseal region contains both cortical and trabecular bone. The cortical bone is formed by condensation or ‘corticalization’ of trabeculae as they arise from the periphery of the growth plate. Adjacent trabeculae coalesce by bone formation upon their surfaces while more centrally placed trabeculae form the metaphyseal trabecular compartment. The cortico-trabecular transitional region so formed between the cortical bone and the more centrally placed trabecular bone is composed of mineralized bone matrix that transitions from the more compact cortical configuration to a more open spongy trabecular configuration with more void volume (porosity) [20].

The purpose of this study was to quantify any association between bone microstructure and the presence of a stress fracture in the lower limb. We hypothesized that young adult individuals sustaining stress fractures have thinner and more porous cortices and reduced trabecular density characterised by reduced numbers and thinner trabeculae.

## 2. Materials and Methods

This was a cross sectional case control study. Thirty-four patients presenting with acute focal lower limb pain were assessed for possible stress fractures using magnetic resonance imaging (MRI) [21]. Participants were recruited from the osteoporosis out-patients department of the St. Vincent Hospital and the Austrian Military Hospital in Vienna/Austria.

Patients were included if they were above 18 years of age and sustained a recent MRI-diagnosed stress fracture within 14 days prior to inclusion. Patients receiving any medication affecting bone metabolism (parathyroid hormone, intravenous or oral bisphosphonate, strontium ranelate, raloxifene, hormonal replacement therapy or anabolic steroids) were excluded. Other exclusion criteria included the presence of metabolic bone disease, a history of malignancy, hypo- or hyperparathyroidism, pregnancy and lactation.

Of the 34 subjects, 26 were included (11 females, 15 males). The study group included five military recruits, three long distance runners, one sprinter and one professional tennis player, the other 16 patients were recreational sportsmen and women. We compared these results with data obtained from 62 healthy age- and sex matched Caucasian controls (42 Austrian subjects, 20 Australian subjects). The study was approved and supervised by an independent local ethics committee in Vienna/Austria.

### 2.1. Magnetic Resonance Imaging

Examinations were performed on 1.5 or 3.0 T scanners (Signa HDx, GE Healthcare, USA; Intera, Philips, The Netherlands, Avanto, Siemens, Germany) using a phased-array extremity coil. All MRI examinations included an axial T1-weighted spin-echo (SE) sequence and an axial fat-suppressed T2-weighted turbo spin-echo (TSE) or a short-tau inversion recovery (STIR) sequence. The presence of bone marrow edema and stress fracture was seen as decreased signal intensity on T1 weighted SE images, and markedly increased signal intensity on STIR images or T2 weighted fat-suppressed TSE images [22]. In 16 patients High Resolution Multi-Detector CT (HR-MDCT) scanning was performed with a 128-row MDCT scanner (Somatom AS+, Siemens, Forchheim, Germany). Images were acquired in ultrahigh resolution (UHR) mode covering the area of interest as defined by MRI. Slice thickness was 0.6 mm, field of view of 160–300 mm (*x*-axis) × 160–300 mm (*y*-axis), with a matrix of 512 × 512, which equates to a resolution of 0.3–0.6 mm (*x*-axis) × 0.3–0.6 mm (*y*-axis) × 0.6 mm slice thickness (*z*-axis). A linear stress fracture was visible in 16 of 26 cases (61.5%) [23]. In 8 patients the fracture was identified in the metatarsals, in 8 patients in the calcaneus and ankle, in 6 patients in the proximal tibia adjacent to the knee, in 2 patients in the femur condyle, and in 2 patients in the hip (one femoral head and one acetabulum).

### 2.2. Measurement of Bone Microarchitecture

**Figure 1:**
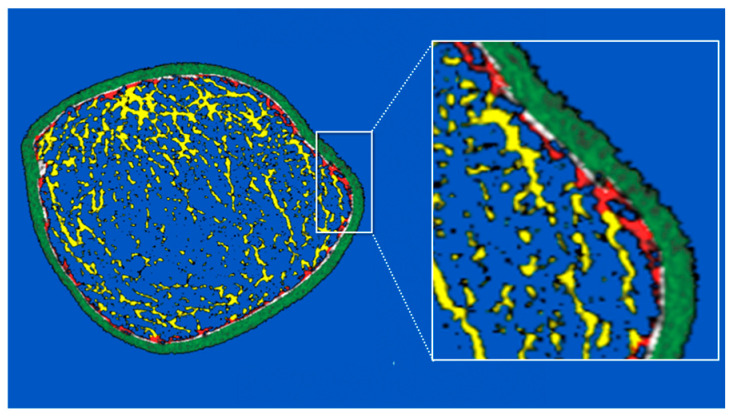
HR-pQCT image of the distal tibia of an 18-year-old male patient with a stress fracture. Compact appearing cortex (green); outer transitional zone (white); inner transitional zone (red); medullary area (yellow).

Scans were obtained from the non-dominant distal radius and unfractured side of the distal tibia using by HR-pQCT scanner (XtremeCT; Scanco Medical AG, Brüttisellen, Switzerland) using the standard in vivo protocol (60 kVp, 900 µA, 100-ms integration time) [24]. StrAx1.0 is a new algorithm that segments bone from background, and then bone into its compact appearing cortex (CC), outer transitional zone (OTZ) and inner transitional zone (ITZ), and trabecular compartments (see Figure 1); and in so doing correctly confines the trabecularized cortex (i.e., cortical fragments) to the transitional zone rather than incorrectly allocating cortical fragments (which look like trabeculae) to the medullary compartment; a segmentation error which overestimates ‘trabecular’ density [25]. The OTZ is the trabecularized cortex adjacent to the CC whereas the ITZ is the trabecularized cortex adjacent to the medullary cavity. The latter also contains true trabecular bone (of growth plate origin).

Of the 110 slices in the region of interest imaged by HR-pQCT the analysis is restricted to the 40 most proximal slices because the 70 distal slices often have very thin cortices, and hence are unsuitable for unambiguous quantification of cortical porosity. Porosity was quantified as previously reported. The coefficient of variation for segmentation and quantification of porosity ranges from 0.54 to 3.98% depending on the compartment [26,27].

### 2.3. Statistical Analysis

Testing for normality of distribution of a variable was conducted using Shapiro–Wilk test. Two sample *t*-test was used to test the difference in mean between cases and controls if the data was approximate normal, otherwise nonparametric Mann–Whitney test was used to test the difference in the medians. Post hoc power calculations were done. Initially, the statistical test was computed without adjustment for other covariates, and then adjustment for age and height was carried out. Spearman’s correlations were used to assess relationships between porosity and medullary CSA/total CSA. Univariate logistic analysis was used to examine the association between porosity of CC, OTZ, ITZ and trabecular volumetric BMD and fracture risk. All variables were standardized to have mean of 0 and SD of 1 and were used to predict the odds ratio (OR). The analyses were performed on data at both tibia and radius site for males and females separately. We used statistical software STATA (StataCorp, 2009), version 11, to conduct all analyses. *p*-values were computed for two-sided tests and values less than 0.05 were considered as significant.

## 3. Results

Characteristics of the entire study population are described in Table 1.

### 3.1. Males

As shown in Figure 1 and Table 2, relative to controls, at the distal radius, cases had smaller cortical CSA (*p* = 0.012) due to a smaller CC CSA (*p* = 0.005) and a smaller OTZ CSA (*p* = 0.003). Cases had higher porosity of the OTZ (*p* = 0.006), ITZ (*p* = 0.043) and CC (*p* = 0.023). Cortical vBMD was lower (*p* = 0.002). Trabecular vBMD was lower (*p* = 0.002). Trabecular thickness was reduced (*p* = 0.001), connectivity was reduced (*p* = 0.012) and separation increased (*p* = 0.007) (Figure 1, Table 2).

At the distal tibia, relative to controls, cases had a smaller cortical CSA (*p* = 0.008) due to smaller CC CSA (*p* = 0.015) and a smaller OTZ CSA (*p* = 0.012). Porosity was not higher at this site. Cortical vBMD was lower (*p* = 0.016). Trabecular vBMD was lower (*p* = 0.001). Trabecular thickness was reduced (*p* = 0.0004), connectivity was reduced (*p* = 0.006) and separation increased (*p* = 0.007) (Table 2).

### 3.2. Females

The results were similar in females. Relative to controls, at the distal radius, cases had significant higher porosity of the OTZ (*p* = 0.028) and ITZ (*p* = 0.030), but porosity was not significantly higher at CC (*p* = 0.054). Cortical vBMD was not lower, but trabecular vBMD was lower (*p* = 0.041). At the distal tibia, porosity was higher in the OTZ (*p* = 0.035) and ITZ (*p* = 0.009) but not at the CC. Cortical and trabecular vBMD were not lower. No differences in trabecular morphology were detected at either site (Table 2).

**Figure 2:**
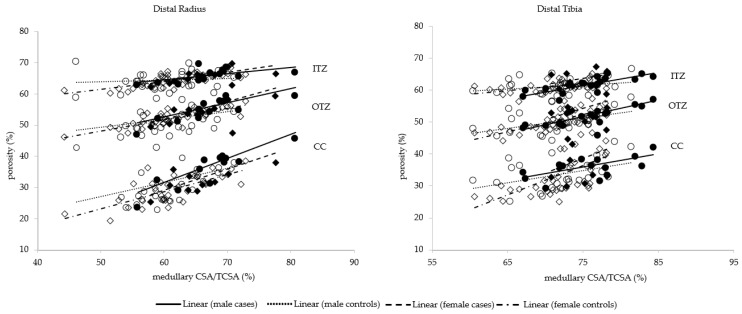
Porosity of the compact appearing cortex (CC), outer and inner transitional zones (OTZ, ITZ) as a function of medullary cross-sectional area (CSA)/total CSA at the distal radius and distal tibia in male cases (filled dots), male controls (open dots), female cases (filled squares) and female controls (open squares). All *p* < 0.001 except for the distal radius ITZ in males (*p* = 0.004).

**Figure 3:**
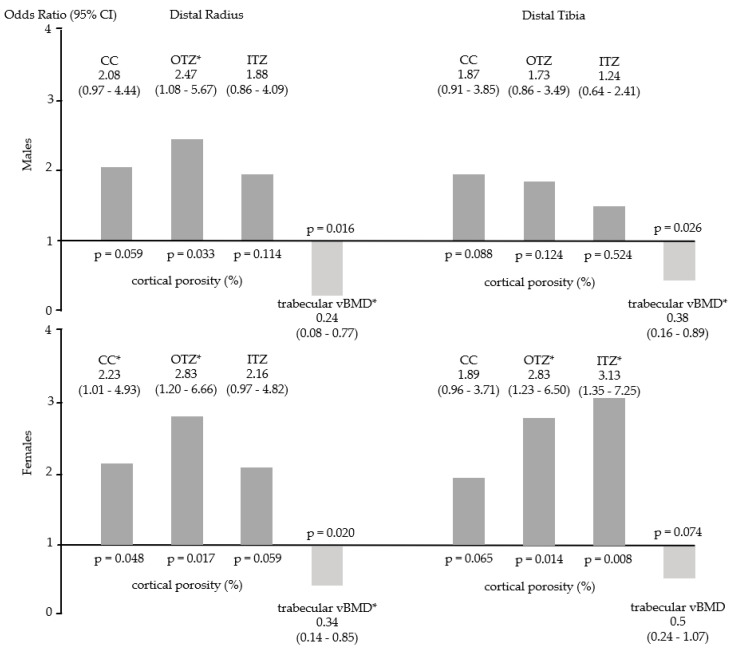
Odds ratio (OR) for fracture (mean and 95% Confidence Intervals, CI) for porosity of the compact appearing cortex (CC), outer and inner transitional zones (OTZ, ITZ) and trabecular volumetric bone mineral density (vBMD) at distal radius and distal tibia in males and females. * = significant *p*-values.

In both sexes, at both sites, associations were detected between porosity of CC, OTZ and ITZ and medullary CSA/TCSA (*p* < 0.001, except for the distal radius ITZ in males with *p* = 0.004) (Figure 2). Cortical porosity was associated with an increased odds of stress fracture, with ORs ranging from 1.24 to 3.13 depending on the cortical compartment, though not all sites demonstrated a statistically significant increase in odds. Higher trabecular vBMD was protective, demonstrating a statistically significantly lower odds of fracture at the distal radius in males and females and the distal tibia in males (Figure 3).

## 4. Discussion

We report that men and women with stress fractures had increased porosity, observed in the inner and outer cortico-trabecular transitional zones of the distal radius in both sexes and distal tibia in female cases. Porosity of the compact appearing cortex was increased only in males at the distal radius. Odds ratio for a stress fracture was associated with increased porosity of the outer cortico-trabecular transitional zone in three of four locations. Cortical vBMD, which in part, reflects porosity, was also reduced. Furthermore, trabecular vBMD was lower due to reduced trabecular thickness, not numbers. Males with stress fractures had thinner trabeculae with greater separation.

Stress fractures in military cadets and athletes have been subject of most studies using dual x-ray absorptiometry (DXA), x-ray and single photon absorptiometry or nuclear bone scanning [11,12,13,14,28]. Fewer studies have used peripheral quantitative computed tomography (pQCT), or HRpQCT to describe bone characteristics in association with stress fractures [10,17,29].

Our findings confirm some, not all previous studies. For example, Beck et al., reported thinner cortices in female cadets and narrower subperiosteal diameters in male cases [12]. Reduced cortical area was also reported by Popp et al., in runners with a stress fracture [30]. While the finding of greater total bone cross sectional was not statistically significant in our study, Weidauer et al., also reported greater periosteal circumference in athletes with a stress fracture [31]. Among military recruits and male runners, Giladi et al., and Popp et al., reported reduced tibial cross sectional area, while HRpQCT-analyses of our cases and work by Schanda et al., reported larger tibial and radial cross sectional area [14,17,29]. These subjects also had reduced cortical vBMD, reduced cortical CSA with increased cortical porosity. In both studies, and work by Schnackenburg et al., trabecular vBMD was found to be reduced [10].

While bone loss cannot be excluded as a cause of these deficits, 20 of the 26 subjects were under 50 years of age. Therefore, higher porosity in the cortico-trabecular junctional zone, with less consistently elevated porosity of the compact cortex, and deficits in trabecular bone may have their origin in the growth-related assembly of bone in some subjects rather than its age-related deterioration [18]. We cannot distinguish these alternatives in a cross sectional study.

We suggest that the thinner and more porous metaphyseal cortex in cases is, in part, the result of a reduction in bone formation upon trabeculae emerging from the periphery of the growth plate. These may fail to coalesce leaving a thinner and more porous cortex (failed corticalization of trabeculae). Reduced bone formation upon trabeculae emerging from the centre of the growth plate may result in the lower metaphyseal trabecular density [32]. This is supported by the presence of a smaller and more porous OTZ suggesting impairment of coalescence as thinner and more separated trabeculae fail to coalesce leaving larger pores adjacent to CC. The more porous cortex will also be thinner due to failure of cortical thickening taking place by adsorption of trabeculae upon the endocortical surface. The thinner cortices are unlikely to be due to reduced periosteal apposition because total CSA was not reduced. Less corticalization of trabeculae may also be partly responsible for the relatively larger medullary canal area as adsorption of trabecular bone upon the endocortical surface is partly responsible for reducing medullary canal area from the ‘inside’.

The relatively larger medullary canal area, thinner cortices and higher porosity in the cases may also be due to greater growth-related endocortical resorptive modeling excavating a larger medullary canal during growth and concurrent intracortical resorptive remodeling forming osteons, each with their central Haversian canal. The cases were slightly taller and had wider bones. Taller persons assemble their wider bones by excavation of a disproportionately larger medullary canal such that the wider bone has a thinner cortex relative to its total CSA [19]. Wider bones are assembled with less material relative to their size because resistance to bending is a fourth power function of their radius–less material is needed to achieve a given bending strength than is needed in a narrower bone [33,34].

Wider bones may also be more porous. The greater amount of modeling required to assemble a larger total and medullary CSA is accompanied by more intracortical remodeling forming secondary cortical osteons, each with their Haversian canal (which forms most ‘porosity’ as seen in cross section) [35]. This is suggested by the positive correlation between medullary canal area and porosity reported here, and elsewhere [19]. This correlation is consistent with the notion of coordinated assembly of the external size, shape and internal architecture of bone by periosteal, intracortical, endocortical surface dependent modeling and remodeling [36].

The increased risk for fracture reported in taller persons is inconsistent with greater resistance to bending observed in wider bones [19]. However, the greater porosity and relatively thinner cortices of wider bones may offset the advantage of greater width. Resistance to bending is a 4th power function of the distance a unit volume of bone is placed from the neutral axis of a long bone [33]. However, resistance to bending is a 7th power function of cortical porosity and a 3rd power function of trabecular density, so the deleterious effect of microstructural abnormalities may offset any benefit achieved by greater bone width [37]. The ability of bone to deform without cracking decreases as porosity increases [37,38]. The porosity may form stress concentrators predisposing a higher risk to fracture following repetitive loading [39]. Additionally, porosity may reduce compressive strength as the cross-sectional area of the cortex is less mineralized bone matrix and more void area [38].

Both deterioration in cortical and trabecular bone may result from impaired growth, particularly because cortical bone at metaphysis is partly formed by condensation of trabeculae in the periphery of the growth plate to form the cortex while trabeculae emerging from the center of the growth plate form metaphyseal trabecular bone.

Bone loss contributing to the deficits cannot be excluded but appears to be less likely given the age of the cases. Remodeling initiated upon Haversian canal surfaces excavate resorptive cavities enlarging the canal focally producing higher intracortical porosity and stress concentrators predisposing to micro cracks in cortical bone [39]. Even though remodeling may be still balanced in young adults, the refilling phase takes about 3 months, so resorption cavities upon trabeculae surfaces form stress concentrators which may predispose to microdamage in the face of repetitive strain [40]. The repetitive strains lead to accumulation of unrepaired microdamage ultimately producing a stress fracture [41,42,43].

This study has several limitations. First, it was a cross-sectional case control study. We cannot distinguish whether the porosity contributed to the stress fracture or whether the sequence of events was the reverse. However, measurements were performed within 14 days after injury on the contralateral side, which makes the latter less likely. Most of the cases were recreational sportsmen and women as were controls. We have no data concerning daily physical activity of the controls. Second, most of the male controls were from Australia. However, results were similar in females. Female cases and controls were Austrian. Third, the small number of cases, particularly in females, may have limited the power to achieve statistical significance of differences between cases and controls. Further investigation with a prospective study design and larger sample size are needed. The stress fractures in the cases were diagnosed in their lower limbs whereas differences in bone microstructure were measured at the distal tibia and the distal radius [26,44,45,46]. Despite this limitation Mikolajewicz et al., and others have reported fracture prediction using HRpQCT. [24,47,48]. Finally, the contribution of pore size and pore number to the reduction in total porosity was not evaluated.

## 5. Conclusions

In summary, stress fractures are common among young adults and are likely to be partly the result of deficits in cortical and trabecular bone microstructure. Whether these deficits have their origin established during growth, during advancing age or both requires further study.

## Figures and Tables

**Table 1:** Characteristics of cases and controls.

Males	Cases (15)	Controls (24)	*p*
Age [ys]	29.2 ± 2.6	30.9 ± 1.7	0.450
Weight [kg]	77.5 ± 3.5	84.1 ± 3.1	0.180
Height [m]	1.81 ± 0.02	1.78 ± 0.02	0.140
Females	Cases (11)	Controls (38)	*p*
Age [ys]	47.8 ± 4.7	40.5 ± 2.3	0.140
Weight [kg]	68.5 ± 5.4	61.8 ± 1.2	0.340
Height [m]	1.67 ± 0.02	1.65 ± 0.01	0.290

Results are shown as mean ± SE (Standard error of mean); *p*-value comparing cases and controls (*p* < 0.05).

**Table 2:** Comparison of microarchitecture of the distal radius and distal tibia in cases and controls.

	Distal Radius						Distal Tibia					
	Males			Females			Males			Females		
	Cases (15)	Controls (24)	*p*	Cases (11)	Controls (38)	*p*	Cases (15)	Controls (24)	*p*	Cases (11)	Controls (38)	*p*
TCSA [mm^2^]	314 ± 25	287 ± 14	0.418 **	225 ± 8.1	203 ± 5.9	0.878 *	820 ± 54	762 ± 34	0.841 **	625 ± 19	603 ± 16	0.768 *
Medullary CSA [mm^2^]	215 ± 24	177 ± 11	0.685 **	149 ± 7.7	126 ± 5.3	0.655 *	629 ± 52	553 ± 32	0.714 **	467 ± 16	437 ± 15	0.941 *
Medullary CSA/TCSA	67.1 ± 1.7	60.9 ± 1.2	0.006 *	66.0 ± 1.7	61.4 ± 1.0	0.161 *	75.8 ± 1.4	71.8 ± 1.1	0.059 *	74.5 ± 0.8	71.9 ± 0.7	0.326 *
Cortical CSA [mm^2^]	98.7 ± 2.4	110 ± 4.3	0.012 *	76.0 ± 3.8	76.6 ± 1.5	0.363 *	190.3 ± 6.4	208.7 ± 6.0	0.008 *	159 ± 5.9	166 ± 2.6	0.272 *
CC CSA [mm^2^]	62.8 ± 1.8	73.4 ± 3.2	0.005 *	49.6 ± 3.0	49.7 ± 1.2	0.414 **	125 ± 5.9	139 ± 5.3	0.015 *	104 ± 5.2	108 ± 2.2	0.590 *
OTZ CSA [mm^2^]	8.12 ± 0.2	9.86 ± 0.5	0.003 *	6.63 ± 0.5	6.47 ± 0.2	0.320 **	17.5 ± 0.9	20.9 ± 1.1	0.012 **	13.8 ± 0.8	14.9 ± 0.4	0.349 *
ITZ CSA [mm^2^]	27.8 ± 1.4	26.4 ± 1.4	0.616 *	19.8 ± 0.9	20.4 ± 0.7	0.139 *	48.9 ± 2.9	49.0 ± 1.9	0.512 *	41.4 ± 1.8	43.5 ± 1.5	0.182 *
Cortical CSA/TCSA	32.9 ± 1.7	39.1 ± 1.2	0.006 *	34.0 ± 1.6	38.6 ± 0.9	0.161 *	24.2 ± 1.4	28.2 ± 1.1	0.059 *	25.5 ± 0.8	28.1 ± 0.7	0.326 *
Porosity CC [%]	37.3 ± 2.0	32.2 ± 1.5	0.023 *	33.4 ± 1.8	29.6 ± 0.7	0.054 *	36.4 ± 1.1	33.7 ± 0.9	0.090 *	38.4 ± 2.2	33.8 ± 1.1	0.295 **
Porosity OTZ [%]	55.9 ± 1.2	52.0 ± 0.9	0.006 *	55.2 ± 1.1	52.3 ± 0.4	0.028 **	52.2 ± 0.9	50.4 ± 0.7	0.058 *	54.0 ± 1.0	50.8 ± 0.5	0.035 **
Porosity ITZ [%]	66.0 ± 0.6	64.4 ± 0.6	0.043 *	65.8 ± 0.7	64.3 ± 0.3	0.030 *	61.8 ± 0.7	61.3 ± 0.5	0.245 *	63.5 ± 0.7	61.4 ± 0.3	0.009 *
Total vBMD [mgHA/cm^3^]	332 ± 16	412 ± 13	0.001 *	309 ± 21	369 ± 12	0.061	310 ± 15	366 ± 11	0.002 *	266 ± 12	311 ± 8.5	0.104 *
Cortical vBMD [mg HA/cm^3^]	657 ± 20	731 ± 16	0.002 *	684 ± 20	721 ± 9.9	0.175 *	674 ± 13	717 ± 11	0.016 *	630 ± 20	684 ± 12	0.106 *
Tr. vBMD [mg HA/cm^3^]	168 ± 5.9	205 ± 8.9	0.002 *	113 ± 11	144 ± 5.4	0.041 *	192 ± 9.5	225 ± 8.7	0.001 *	141 ± 8.7	1612 ± 5.4	0.354 *
Tr. thickness [mm]	0.07 ± 0.003	0.10 ± 0.004	0.001 *	0.05 ± 0.006	0.06 ± 0.003	0.056 *	0.08 ± 0.005	0.1 ± 0.004	0.0004 *	0.06 ± 0.004	0.07 ± 0.003	0.332 *
Tr. connectivity density [1/mm^2^]	2.89 ± 0.2	3.56 ± 0.2	0.012 *	1.65 ± 0.2	2.16 ± 0.1	0.127 *	3.47 ± 0.2	4.09 ± 0.3	0.006 *	2.27 ± 0.2	2.67 ± 0.1	0.570 *
Tr. separation [mm]	0.89 ± 0.04	0.80 ± 0.03	0.007 *	1.15 ± 0.09	0.96 ± 0.03	0.070 **	0.86 ± 0.04	0.78 ± 0.03	0.007 *	1.06 ± 0.05	1.00 ± 0.03	0.885 *
Tr. number [1/mm^2^]	3.16 ± 0.1	3.25 ± 0.1	0.246 *	2.60 ± 0.2	2.87 ± 0.1	0.320 **	3.67 ± 0.1	3.70 ± 0.1	0.166 *	3.21 ± 0.1	3.29 ± 0.08	0.587 *

Results are shown as mean ± SE (standard error of mean); *p*-value comparing healthy controls and fracture patients, adjusted for age and height, using * two-sample *t*-test and ** nonparametric Mann–Whitney test on the residuals of regression of a variable on age and height (*p* < 0.05). TCSA = total cross sectional area, CSA = cross-sectional area; CC = compact-appearing cortex; OTZ = outer transitional zone; ITZ = inner transitional zone; vBMD = volumetric bone mineral density; Tr = trabecular; HA = hydroxyapatite.

## Data Availability

The data presented in this study are available on request from the corresponding author.
